# Active participation of patients with type 2 diabetes in consultations with their primary care practice nurses – what helps and what hinders: a qualitative study

**DOI:** 10.1186/s12913-019-4572-5

**Published:** 2019-11-08

**Authors:** E. du Pon, A. T. Wildeboer, A. A. van Dooren, H. J. G. Bilo, N. Kleefstra, S. van Dulmen

**Affiliations:** 1 Research Group Process Innovations in Pharmaceutical Care, Utrecht University of Applied Sciences, PO Box 12011, 3501 AA Utrecht, the Netherlands; 2 Diabetes Centre, Isala, Zwolle, Dr. Spanjaardweg 11, 8025 BT Zwolle, the Netherlands; 3 Department of Health Science, Section of Nursing Research, University of Groningen and University Medical Center Groningen, Hanzeplein 1, 9713 GZ Groningen, the Netherlands; 4 Department of Internal Medicine, University of Groningen and University Medical Center Groningen, Hanzeplein 1, 9713 GZ Groningen, the Netherlands; 5 Medical Research Group, Langerhans, Ommen, the Netherlands; 6 Nivel (Netherlands institute for health services research), Otterstraat 118, 3513 CR Utrecht, the Netherlands; 7 Radboud Institute for Health Sciences, Department of Primary and Community Care, Radboud university medical center, Nijmegen, the Netherlands; 8 Faculty of Health and Social Sciences, University of South-Eastern Norway, Drammen, Norway

**Keywords:** Patient participation, Type 2 diabetes, Practice nurse, Diabetes care, Primary care, Patient–nurse communication

## Abstract

**Background:**

Patients with type 2 diabetes mellitus (T2DM) receiving primary care regularly visit their practice nurses (PNs). By actively participating during medical consultations, patients can better manage their disease, improving clinical outcomes and their quality of life. However, many patients with T2DM do not actively participate during medical consultations. To understand the factors affecting engagement of patients with T2DM, this study aimed to identify factors that help or hinder them from actively participating in consultations with their primary care PNs.

**Methods:**

Two semi-structured focus groups and 12 semi-structured individual interviews were conducted with patients with T2DM (*n* = 20) who were undergoing treatment by primary care PNs. All interviews were transcribed verbatim and analyzed using a two-step approach derived from the context-mapping framework.

**Results:**

Four factors were found to help encourage patients to actively participate in their consultation: developing trusting relationships with their PNs, having enough time in the appointment, deliberately preparing for consultations, and allowing for the presence of a spouse. Conversely, four factors were found to hinder patients from participating during consultations: lacking the need or motivation to participate, readjusting to a new PN, forgetting to ask questions, and ineffectively expressing their thoughts.

**Conclusion:**

Patients lacked the skills necessary to adequately prepare for a consultation and achieve an active role. In addition, patients’ keen involvement appeared to benefit from a trusting relationship with their PNs. When active participation is impeded by barriers such as a lack of patient’s skills, facilitators should be introduced at an early stage.

**Trial registration:**

Current Controlled Trials NTR4693 (July 16, 2014).

## Background

Type 2 diabetes (T2DM) is a chronic condition associated with adverse lifestyle habits. The rapidly increasing prevalence of T2DM in the Netherlands has reached 65 per 1000 inhabitants of all ages and 230 per 1000 inhabitants aged 65 years or older [1]. This rate of occurrence is expected to increase by another 30% by 2030 [1]. The success of T2DM treatment largely depends on the patient’s ability and willingness to self-manage the disease in daily life, including following advice and adhering to medication [2]. Patients with T2DM regularly visit health care providers (HCPs). These visits are intended to monitor their medical condition and support self-management. Higher levels of patient participation in these consultations have been shown to stabilize blood glucose and improve quality of life [3–5]. Participation in medical consultations refers to actively contributing to the care process by, for example, asking questions, expressing concerns, and stating preferences [6]. As part of patient-centered care [7, 8], patient participation is also a prerequisite for shared decision-making (SDM), which has been demonstrated to improve clinical, psychosocial, and behavioral outcomes [9]. The complexities of changing behavior in T2DM require a counseling-based approach rather than the traditional approach of providing information and advice [10, 11].

In the Netherlands, patients with T2DM are primarily treated in primary care by general practitioners (GPs) and their practice staff, including practice nurses (PNs). PNs conduct routine diabetes consultations, including physical examinations, blood glucose checks, and other laboratory tests, every 3–6 months and discuss the results with patients. PNs also inquire about a patient’s well-being, occurrence of hypoglycemia or hyperglycemia, lifestyle issues, and adherence to medication. The diabetes care provided by a PN is comparable to that provided by a GP; both GPs and PNs perform lifestyle counseling according to generally acknowledged criteria [12, 13].

Thus far, patients have generally not evolved into proactive health care consumers during medical consultations [14–18]. These consultations frequently do not address the use of medication, medication side effects, and lifestyle issues [15, 17]. A recent study by Henselmans et al. (2015) revealed that many patients with chronic conditions expressed an interest in communication support [19]. However, patients were reportedly hindered in their communication because they feared being seen as bothersome, perceived time pressure during the visit, and experienced difficulty remembering topics post-visit [19].

Although some previous research on barriers in communication exists, it specifically focused on patients with other chronic conditions [20, 21], patients with chronic conditions in general [19, 20, 22, 23], or patients with T2DM in countries outside of the Netherlands [10, 24]. Consequently, participation of patients with T2DM during PN consultations was not investigated in the Dutch situation before. Therefore, this study aimed to identify the factors that help and hinder active participation of patients with T2DM in consultations with their primary care PNs. These findings may provide evidence for designing interventions aimed at improving patient participation.

## Methods

This study adopted a qualitative design and was registered with the Medical Ethics Committee of Isala (Zwolle, the Netherlands; METC no. 14.07104). The committee declared that the study did not fall under the scope of the Medical Research Involving Human Subjects Act [25, 26].

### Participants

Primary care treated patients (18 years and older) diagnosed with T2DM were included in this study. Due to their important role in diabetes care, spouses or relatives of the patients were also invited to participate in the interviews [27–29]. A lifestyle-dependent condition such as T2DM is known to influence the daily lives of a patient’s spouse, so PNs often invite them to join consultations [10].

### Recruitment

In May 2015, all general practices in the Zwolle region of the Netherlands were informed of the study and asked for permission to recruit patients from their practices. In case of permission, the general practices allowed Medrie Care Group in Zwolle to recruit during retinopathy screenings. These screenings are a part of the annual T2DM checkups. Patients were recruited at the Medrie Care Group using flyers and posters. Since all patients with T2DM in the Zwolle region (approximately 12,000) are usually scheduled for these screenings, this recruitment method was expected to result in a representative sample.

During a four-week recruitment period, two practice assistants with experience in performing the retinopathy screening introduced the study to eligible patients. After the screening, each patient interested in participating was enrolled in the study and signed an informed consent form. Subsequently, these patients, and any partners or relatives, were invited by telephone to a focus group at the Medrie Care Group in Zwolle in July 2015. Those who were unable to travel to the focus group location or did not feel comfortable speaking in a group were invited to participate in individual interviews held at their homes in September and October 2015.

### Study design

In this study, a two-step approach [20] derived from the context mapping framework was followed [30].

#### Step 1: sensitizing

Sensitizing is a process that triggers, encourages, and motivates patients to think, reflect, wonder, and explore aspects of their personal contexts in their own time and environment [30]. This step was intended to enhance the quality and quantity of the patients’ contributions in later individual or focus groups [31]. To encourage patients to consider the interview topics in advance, they were invited to formulate their experiences, preferences, and needs and reflect upon them. One week prior to the interviews, the patients received a small booklet with open-ended questions regarding their consultations with their PNs. The questions were divided into the following themes: T2DM diagnosis, discussion about T2DM, consultation with the PNs, and self-reflection. Table 1 presents the booklet questions, which were based on a booklet used in a previous study [20]. To help patients answer the more abstract questions, example answers and small stickers of relevant pictures and words to use as starting points were included. In addition, patient characteristics (sex, age, level of education [low, middle, or high], diabetes duration, familiarity with their PN [slightly, moderately, or very], and type of treatment [lifestyle only, oral medication, or insulin]) were collected. We asked each patient to bring the completed booklet to the individual or focus group. The main objective of this sensitizing process was to promote self-reflection on the part of the patients.

**Table 1:** Questions asked in the booklets and during the interviews

Themes	Questions
*Booklets*	
*Characteristics*	Sex, age, education, diabetes duration, familiarity with PN, type of treatment.
*T2DM diagnosis*	Can you say something about the moment you heard you have diabetes and what this meant to you?
*Discussing T2DM*	Who are the three most important people for you to talk to about your diabetes (including at least one care provider)? How do you experience communicating with them?
*Consultations with the PN*	Can you say something about the proceedings of your consultations with the practice nurse? (For example: Is there a clear conversation structure? Do you make notes? Do you ask many questions, not that many, or none? If you bring a companion to the consultation, does this person ask questions?)
	Can you describe your attitude during the consultations?
*Self-reflection*	Are there issues you would like to change for yourself before, during, or after a consultation? Are there issues you have already changed regarding your consultations? (For example: Do you prepare for your consultations differently than in the past? Do you ask more, fewer, or other types of questions now than in the past?)
*Interviews*	
*Preparation*	Do you prepare for consultations with your practice nurse? If so, how?
*Asking questions*	Do you ask questions during consultations? If so, do you often know which questions you need to ask? After the consultations, do you feel that your questions were answered? If you have not asked your practice nurse everything you wanted to ask, what are your main reasons?
*Problems*	Do you sometimes experience problems talking with your practice nurse? Why? Why not?
*Active role*	Do you want to participate more actively during the consultations? If so, why? What do you hope to achieve?
*Support*	What kinds of support could help you during consultations with your practice nurse? How can the practice nurse help you achieve that support?

Abbreviations: *PN* practice nurse, *T2DM* type 2 diabetes

#### Step 2: individual interviews and focus groups

In the second step, the patients engaged in semi-structured 45-min individual interviews or semi-structured 90-min focus groups. Individual interviews allow for a great amount of time and attention to be devoted to a single patient, which often leads to detailed information [30]. Moreover, focus groups enable patients to react to each other’s experiences, convey a global view of the topic, and exchange diverse amounts of information [30]. This is expected to lead to deeper insights than individual interviews, in which the patient is less challenged to consider other perspectives.

Permission was requested to audio record the individual interviews and video record the focus groups. Video recordings were necessary for the focus groups, as they facilitated the transcription of the patients’ responses. The audio and video recordings will be stored for up to 15 years at Utrecht University of Applied Sciences.

First, the interviewer (EdP) introduced the study by explaining her interest in patient participation in diabetes consultations with their PNs. She further explained that the patients’ experiences could help researchers, HCPs, and other patients better understand factors that help or hinder patient participation. The interviewer also emphasized that the PNs would not be judged, as the aim of the interviews was to share experiences anonymously.

Next, the interview started with general questions concerning patient perceptions about their own role during consultations with their PNs. The interviews continued with questions asking what helped and what hindered their participation in these consultations. The interview themes were adopted from empirical literature (inductive). The same topics were discussed and the same questions were asked for both the individual interviews and the focus groups. Table 1 presents the interview questions.

### Data analysis

The framework method developed by Ritchie and Lewis [32] was applied for data analysis. This systematic and flexible approach to analyzing qualitative data is most commonly used for the thematic analysis of semi-structured interview transcripts and produces highly structured summarized data as an output. The framework provides seven clear steps, which are presented below [33, 34].

#### Step 1: transcription

The video and audio recordings were anonymously transcribed verbatim by three students. The transcripts included large margins and adequate line spacing for subsequent coding and notes.

#### Step 2: familiarization with the interviews

This stage was particularly important since students transcribed the individual interviews and focus groups, while EdP was responsible for analyzing the data. EdP familiarized herself with the individual interviews and focus groups by repeatedly listening to and watching the recordings.

#### Step 3: coding

EdP read the transcripts carefully and highlighted certain codes, such as salient or frequently mentioned words, sentences, or phrases, in both the transcriptions and the comments from the booklets. Coding was intended to classify all data in order to systematically compare it with other parts of the data set. New codes emerged from the process of data analysis (deductive). The MAXQDA software program (VERBI Software GmbH, Berlin, Germany) facilitated the coding process [35]. MAXQDA is designed for computer-assisted qualitative and mixed methods data, text, and multimedia analysis in academic, scientific, and business institutions [35]. The program was used for the organization and analysis of our data, such as the import of transcriptions; reading, editing and coding data; searching and tagging words; and annotating data. MAXQDA also recognizes different speakers of focus groups automatically. Therefore, we could easily compare their contributions and analyze each speaker separately.

#### Step 4: developing an analytical framework

After coding the first transcripts, an analytical framework in which codes were placed within categories (e.g., “support of a spouse” and “attitude during the consultation”) was developed. Categories were clearly defined, and several iterations of the analytical framework were required before additional codes no longer emerged.

#### Step 5: applying the analytical framework

Next, EdP and a diabetes specialist nurse (AW) applied and refined the coding framework while independently coding the interviews. When this coding framework was used to define every (sub) code, only fragments of texts concerning the helping and hindering factors associated with the consultation participation were coded. The MAXQDA program expedited this process and ensured that data were easily retrievable at the later stages [35]. Both researchers coded all interviews to determine interrater reliability, and the researchers also discussed new codes and unclear fragments. Credibility was established by discussing the framework and coding with a senior researcher (SvD). A qualitative study is considered credible if its descriptions of human experience are immediately recognized by individuals who share the same experience [33].

#### Step *6: Charting data into the framework matrix*

Reducing data is a vital aspect of the analytical process, during which the original meanings and “feel” of the respondents’ words should remain intact. A spreadsheet was used to generate a matrix into which the data were charted. The data of each transcript were summarized by category and references to quotes were included.

#### Step *7: Interpreting the data*

During this step, EdP and AW noted and discussed interesting ideas, concepts, potential themes, and early interpretations. Since the data seemed complete and useful in answering the research question, particular cases were described to illuminate the patients’ perceptions of when active participation occurred and why. Patient quotes that illustrate the results were retrieved from the interviews and translated from Dutch to English by EdP and verified by a native speaker. The respondents’ names in this article are all fictitious to protect their privacy.

Results were classified using the Feldman-Stewart framework for patient–professional communication within the health care setting. This framework suggests that the patients’ attributes in consultations with their HCPs are influenced by their needs, beliefs, values, skills, and emotions [36]. The communication is directly a function of the attributes, or qualities, of each person involved. The framework can help guide assessing the communication process, in particular how the patient interact in the setting of diabetes consultation.

## Results

### Respondent and interview characteristics

Forty patients were approached as potential participants, 13 of whom withdrew before the start of the study due to logistic reasons (e.g., illness, moving houses, or a hospital visit), and five did not meet the inclusion criteria (see Section 2.1). Of the remaining 22 patients, two did not attend any interview. In total, 20 patients were included in the study, and the mean age of the patients was 71.5 years. All 20 patients had different PNs. In three cases (one men, two women), a spouse accompanied the patient during the interview. Other patient characteristics are presented in Table 2.

**Table 2:** Patient characteristics

	Participants (*n* = 20)
Men	11
Woman	9
Age, median (IQR)	71.5 (68.0–77.8)
Education (*n*)^a^	
*Low*	6
*Moderate*	11
*High*	3
Diabetes duration	
*< 1 year*	2
*1–5 years*	6
*5–10 years*	7
*> 10 years*	5
Familiarity with their PN	
*Slightly familiar*	3
*Moderately familiar*	3
*Very familiar*	14
Type of treatment	
*Lifestyle only*	5
*Oral medication*	12
*Insulin*	3

Abbreviations: *IQR* interquartile range, *PN* practice nurse

^a^Low = no education or primary education; moderate = lower secondary education, (upper) secondary education or post-secondary non-tertiary education (including vocational education); high = tertiary education (bachelor’s degree or higher)

All 20 enrolled patients completed their booklets before the interview. Of these patients, 12 participated in focus groups, and 8 had individual interviews. One focus group consisted of seven patients plus 2 spouses, and the other consisted of 5 patients plus 1 spouse. As the last three interviews did not provide any new information, it was concluded that data saturation had been reached. This means that we reached a point in our analysis of data that sampling more data would not lead to more information related to the research question [37]. The results concerning helping and hindering factors are presented in Table 3.

**Table 3:** Helping and hindering factors in participation reported by patients, classified by the Feldman-Stewart framework

	Helping factors	Hindering factors
Need	Trusting relationship with a PN	Readjustment to a new PN
	Presence of a spouse	–
Belief	Availability of time	Insufficient need or motivation to participate
Value	–	–
Skill	Preparation for the visit	Forgetting to ask questions Expressing thoughts ineffectively
Emotion	*–*	*–*

### Factors helping with active participation

#### Trusting relationship with the PN

Patients perceived a trusting relationship with their PNs as a facilitating factor for active participation. In general, patients experienced their PNs’ manners of communication as pleasant and warm, which encouraged them to discuss their emotions and concerns. For example, patients felt welcome to discuss problems with adhering to a diet or fear of dealing with complications in the future. One patient explained this trust with an example:

*“I was open to her about my candy-eating habits when my weight was too high. When you know each other well, you start to open up more. This process went smoothly; I trusted her soon enough.” (aged 68, very familiar with PN)*

In a trusting relationship, patients also felt encouraged to discuss issues unrelated to their T2DM, such as their personal situations.

*“We talk about the grandchildren we both have, small talk. That has nothing to do with the disease. It is personal. She told me how happy she was when she first became a grandmother. You continue on that subject, and I liked that as well!” (aged 77, very familiar with PN)*

Along with personal situations, emotional events in their lives or in the PNs’ lives were discussed. Patients indicated that sharing emotional events led to a trusting relationship, which strengthened over time.

#### Presence of a spouse

Patients who preferred to visit their PNs together with their spouses indicated that they were supported by their spouses because the latter were already familiar with T2DM.

*“We visit the practice nurse together. That’s nice because my wife has a longer record of dealing with practice nurses than I do, and she knows how they work.” (aged 80, very familiar with PN)*

Other patients reported that their spouses were more accurate in asking questions or that they considered their spouses to be more careful and precise when asking questions about the disease. One patient spoke about the benefit of having a spouse at an appointment:

*“We always go together, as she needs her blood pressure and COPD checked. So, we combine our visits. I find it convenient that she is there with me because I am messy, and she is more precise. I am lazy; why should I know something if somebody else already knows?” (aged 78, very familiar with PN)*

#### Availability of time

Patients indicated that when they did not experience time pressure, active participation was stimulated. The PNs had sufficient time to discuss all necessary subjects, and the patients felt free to ask questions. Thus, questions arose automatically.

According to the patients, the PNs verified whether patients understood them and stimulated patients to ask additional questions or ask for clarification.

*“She checks if you understood; and if not, you can ask again. I certainly do that. I ask something that is unclear to me, and then I will ask again.” (aged 73, very familiar with PN)*

In addition, when patients felt there was enough time, they usually felt welcome to raise (new) topics, even though these discussions might deviate from the PNs’ routines.

*“The consultation can turn at any given time. For example, when my weight is too high, you might start a conversation about the possible cause.” (aged 77, very familiar with PN)*

#### Preparation for the visit

Patients mentioned personal preparation as beneficial in terms of allowing them to actively participate in consultations with their PNs. Some patients commonly prepared questions prior to their consultations, while others formulated questions only out of fear of forgetting them. One patient spoke about list making:

*“Making a list of questions is not only a positive influence on me during the consultation; it is something to go by about things I want to say or ask, things that are important to me. That’s why I do it. This way I never get the feeling afterwards I forgot something because the list is my starting point. I would like to discuss several topics, but that list should be addressed anyhow.” (aged 75, very familiar with PN)*

### Factors hindering active participation

#### Readjustment to a new PN

Patients also expressed a need to readjust to new PNs with whom they did not yet have working relationships. In the beginning, patients reported having a wait- and see attitude and had reservations about questioning their PNs. When they had known one another for a longer period, their attitude became more assertive. A patient reported unease with new PNs:

*“It is annoying. You must get acquainted again to see what kind of person she is. See which way the pendulum swings, things like that. During my first consultation with her, I was really quiet. As I visited her more often, I felt more comfortable, and our conversations opened up more. You get to know each other better. I did not feel comfortable at first but did at times. And, when you finally trust her, she is replaced by a colleague.” (aged 56, moderately familiar with PN)*

Some patients described how their PNs used a tone of voice that annoyed them. One patient explained the importance of this tone:

*“My practice nurse first struck me as someone who did not know how to address ‘yet another patient with diabetes.’ I told her I am not a child anymore, she did not need to patronize me. Such a pedantic tone at first. Now, she is just open.” (aged 77, very familiar with PN)*

#### Insufficient need or motivation to participate

Most patients described communication between the PNs and themselves as evenly balanced interactions, while a few patients indicated that the PNs played the leading role. Patients also mentioned that they were satisfied with the division of roles in the consulting room:

*“I consider it to be just a professional relationship. It is her job to lead the consultation. I am comfortable with that. You must keep the roles clear. It is her job,” (aged 78, very familiar with PN)*

This division of roles in the consulting room seemed to develop naturally, and the patients did not feel the need or motivation to participate more actively. The patients did not have to determine the agenda, did not feel the need to take the lead, or did not want to “take all they could get.” Some patients blamed this lack of need or motivation on the absence of symptoms of their T2DM; they simply did not feel sick. Other patients blamed the mandatory nature of the consultations:

*“I go to the appointments because I have to; if these were not necessary, I would rather not go.” (aged 45, very familiar with PN)*

Patients reported customarily arriving at the consulting room with a wait-and-see attitude or thinking that they only visited the PNs to hear test results. In addition, patients indicated that positive test results often did not lead to discussions, and the issues on the agenda were usually uncomplicated.

*“The conversations are not complicated, so I will not run into difficulties.” (aged 75, very familiar with PN)*

#### Forgetting to ask questions

The patients reported that they sometimes forgot to ask their questions and stated that this could be due to memory problems caused by their older age. One patient explained this forgetfulness:

*“I tend to forget things, so you talk about different matters. Only afterwards you remember what you had actually wanted to ask.” (aged 70, very familiar with PN)*

Other reasons given for forgetting to ask questions included being distracted or having the consultation take another turn (e.g., to a more social conversation).

*“Then, it actually becomes more pleasant [laughs]. As I said, discussing personal things like becoming a grandmother. And then you sometimes forget your questions, and you later think to yourself, ‘oh, well.” (aged 77, very familiar with PN)*

#### Expressing thoughts ineffectively

The patients occasionally mentioned having trouble expressing their thoughts, which hindered their active participation in the consultations. Patients blamed this feeling on their age and memory loss. Patients also reported that problems in expressing thoughts appeared to be caused by the confusion of having multiple conditions. The difficulties in communicating about their comorbidities deterred patients from bringing up other topics about diabetes:

*“I have several different conditions, and diabetes is one of them. If I start to talk about something, she often replies with ‘you should discuss that with the doctor.’” (aged 50, moderately familiar with PN)*

Finally, patients indicated having problems in stating their preferences for a more tailored consultation.

## Discussion

This qualitative study revealed four helping factors and four hindering factors that may affect active participation of patients with T2DM in consultations with their primary care PNs. First, having a trusting relationship with their PNs encouraged patients to express their emotions and concerns.

Applying the framework of Feldman-Stewart, this positive element could be considered a need. Second, the presence of a spouse (a need) during the consultation could also support participation. Third, the availability of time (a belief) stimulated patients to ask questions. Lastly, preparing for the visit (a skill) helped patients discuss the topics that are important to them. By contrast, the results indicated that participating in consultations could be hindered by the following factors: readjusting to a new PN (a need), lacking the need or motivation to participate (a skill), forgetting to ask questions (a skill), and expressing thoughts ineffectively (a skill).

### Needs

The finding that the presence of a spouse could support participation is not surprising because social support is important in diabetes care [27–29], and the spouses of patients with T2DM play key roles in adhering to and maintaining a healthy diet [28]. Moreover, the presence of a companion was associated with a more task-focused exchange, particularly by the patient [38].

In addition, the barrier of “familiarizing oneself with a new PN” identified in this study suggests that a trusting relationship with a PN, which patients value greatly, is needed to enhance active participation [24].

### Skills

An obstacle identified in the Henselmans et al. study [19] (i.e., difficulty remembering discussion topics after an appointments has ended) is comparable to this study’s hindering factor of “forgetting to ask questions.” However, the other two barriers identified by Henselmans et al. (the desire to avoid being bothersome and perceived time pressure) were not encountered in this study. The discrepancy with the results of Henselmans et al. may be explained by the differences in the study settings. The sample investigated by Henselmans et al [19] consisted of patients with a variety of chronic conditions who usually had a GP or a medical specialist as the primary HCP. The patients in this study, all of whom had been diagnosed with T2DM and were seen in primary care, were treated by PNs. Previous research indicates that patients are more satisfied with the care delivered by PNs than GPs [12].

The patients in this study were primarily older adults—which was not surprising as T2DM primarily affects older adults—and they mentioned that “forgetting to ask questions” could be caused by memory problems. The patients also indicated that “having problems expressing their thoughts” interferes with participation. According to the literature, elderly patients appear to ask fewer questions and obtain less medical information than younger patients [16]. While these issues may be age related, health literacy could also play a role because the study primarily included patients with low or middle levels of education. Patients with low literacy levels feel less confident and perceive more barriers in communication [19]. 

### Beliefs

Although patients with T2DM may have concerns and questions that they rarely discussed with GPs [39], in this study, patients did not report experiencing time pressures and instead felt free to ask questions while interacting with their PNs. This finding could be explained by the fact that consultations with PNs are twice as long as those with GPs [16] and that patient participation is more limited in short consultations [40, 41]. In addition, another explanation could be the fact that most patients in our sample were very familiar with their PN. Since the introduction of PNs in the Netherlands in 1999, patients with chronic conditions have become more satisfied with the care delivered [13]. Many patients with diabetes prefer to be supervised by a PN instead of a GP [42]. because PNs have more time for patients and superior knowledge about diabetes [42]. Patients value the fact that the PNs take them seriously, listen carefully, are open, take sufficient time, and provide adequate advice on how to manage complaints [18, 42]. This could explain why patients did not mention an issue related to the relationship of authority with their PN.

Considering the age of the population, it is difficult to determine the actual statuses of the patients’ health or whether other health problems may be contributing factors. Some patients may view other health problems as graver issues than T2DM. In addition, the absence of disease burden in T2DM, which is particularly relevant for recently diagnosed patients [43], could lead to a lack of motivation and willingness both to adopt an active role during consultations and to accept lifestyle changes [30]. In a study examining the readiness of patients to discuss psychosocial problems with PNs during diabetes consultations, Van Dijk et al. (2016) showed that patients view a consultation primarily as a biomedical check-up [44]. Thus, they did not expect to talk about psychosocial problems with their PN. Similarly, we found that patients who lacked the need or motivation to participate only visited the PNs to be information about test results.

Patient participation in consultations should always be considered in its context. Most barriers that PNs perceive with respect to lifestyle counseling in general practice are at the patient level, such as a lack of motivation to modify their lifestyles or insufficient discipline to maintain an improved lifestyle [45]. In this study, we aimed to take a closer look at the patient perspective; however, PNs also need the skills to engage patients. General practice staff in diabetes care, such as PNs, express positive views towards actively engaging their patients [46], which is an important element of SDM [47]. This attitude may explain why most patients in the present study were satisfied with the current mode of participation in the consulting room. T2DM is a complicated and demanding disease, and improving outcomes requires more than improving patient participation during the consultation with PNs. However, relatively small solutions could significantly contribute to improvements in diabetes care [48].

This study has several strengths. First, a varied sample of patients with T2DM was included; patient demographics varied widely in sex, education level, diabetes duration, and type of treatment. Second, by using more than one interview approach, it was possible to capture a wide variety of information [49]. Although the results of the focus groups and the individual interviews were analyzed separately, the focus groups were particularly useful for cataloguing the range of patients experiences, and individual interviews contributed specific details of these experiences [50]. The combination of individual interviews and focus groups methods was used to strive not only for data completeness and confirmation [51, 52] but also for practical considerations. Offering individual interviews to patients unable or unwilling to attend a focus group may have led to fewer refusals or withdrawals [53], as individuals could choose the method that is most convenient to them. Third, the patients seemed well-prepared during the research interviews. The sensitizing process may have enhanced their contributions during the interviews by facilitating the expression of their thoughts about helping and hindering factors. In the focus groups, patients referred to the stories they wrote down in their booklets, which demonstrates the reflection effect of the sensitizing phase. Fourth, because patient participation is an abstract topic, interacting with other patients and hearing their ideas seemed to be helpful to stimulate patients to talk about their own experiences. The presence of a spouse may also have improved the patients’ willingness to share experiences.

Some limitations must also be mentioned. First, due to the abstract nature of the topic, some patients found the booklet questions difficult to answer. They indicated that they did not have an opinion and had not previously thought about the helping and hindering factors in participation. Second, recall bias might have affected the results because we asked the patients about experiences in the past. Third, although multimorbidity was a contributing factor to the study’s findings, no medical health data were collected on these patients. Fourth, because our respondents came from a region in the eastern part of the Netherlands, it is possible that they do not reflect the entire Dutch population with T2DM, which may affect the external validity of this study. Fifth, because patients were recruited during diabetes retinopathy screenings, patients with diabetes retinopathy treated in the secondary care who have probably more complications were not included. The fact that patients could choose between an individual interview or focus group might have introduced bias.

## Conclusions

Patients’ *needs*, *beliefs*, and *skills* influence their participation during consultations. In particular, patients’ skills with regard to taking the lead, expressing thoughts, and preparing for the visits seem to be lacking. However, a trusting relationship with their PNs, which usually develops over time, can help patients to discuss their emotions and concerns. The majority of patients in the study seldom felt the need to participate more actively. This attitude is most likely due to the perceived absence of disease burden and satisfaction with their current roles in the consulting room.

### Practical implications

This study can inform HCPs and policymakers about how patients with T2DM and other chronic diseases can benefit from consultations with a PN. Practice nurses should take into account the potential lack of skills of their patients. Apparently, patients are satisfied with the current modus of participation in the consulting room. Trusting relationships with their PNs, which are usually developed over time, help them to participate more actively. The importance of trust was supported by patients who reported playing a passive role in consultations with new PNs, demonstrating the need to search for other methods to improve self-management behavior. When a patient and a PN are not familiar with each other, the new relationship requires an extra investment of time. In addition, particularly in general practices with several PNs, patients have a need for a PN “of their own.” Other implications are the potential value of encouraging patients to write down questions in advance of the consultation (using a question prompt sheet) [54] or visiting the PN together with their spouses. Future research should explore how multimorbidity influences active participation during PN consultations. Furthermore, the development of an intervention trial versus usual care could be of interest, where patients in the intervention group receive support to capitalize on helping factors and to handle hindering factors.

## Data Availability

The data sets generated and/or analyzed during the current study are not publicly available because public access was not included in the informed consent.

## Abbreviations

AW: Anita Wildeboer
EdP: Esther du Pon
GP: General practitioner
HCP: Health care provider
PN: Practice nurse
SDM: Shared decision making
SvD: Sandra van Dulmen
T2DM: Type 2 diabetes
WMO: Dutch Medical Scientific Research in Human Act
